# Calcium dobesilate efficiency in the treatment of diabetic kidney disease through suppressing MAPK and chemokine signaling pathways based on clinical evaluation and network pharmacology

**DOI:** 10.3389/fphar.2022.850167

**Published:** 2022-09-08

**Authors:** Bingyu Du, Yanyan Yin, Yuqing Wang, Hui Fu, Helin Sun, Zhaodi Yue, Shaohong Yu, Zhongwen Zhang

**Affiliations:** ^1^ Department of Endocrinology and Metabology, Shandong University of Traditional Chinese Medicine, The First Affiliated Hospital of Shandong First Medical University and Shandong Provincial Qianfoshan Hospital, Jinan, China; ^2^ Department of Rehabilitation Medicine, The Second Clinical Medical College and Medical College, Shandong University of Traditional Chinese Medicine, Jinan, China; ^3^ Department of Rehabilitation Medicine, The Second Affiliated Hospital of Shandong University of Traditional Chinese Medicine, Jinan, China; ^4^ College of Rehabilitation Medicine, Shandong University of Traditional Chinese Medicine, Jinan, China; ^5^ Shandong Key Laboratory of Rheumatic Disease and Translational Medicine, Department of Endocrinology and Metabology, Shandong Institute of Nephrology, The First Affiliated Hospital of Shandong First Medical University and Shandong Provincial Qianfoshan Hospital, Jinan, China; ^6^ The Clinical Medical College, Cheeloo Medical College of Shandong University, Jinan, China

**Keywords:** calcium dobesilate, diabetic kidney disease, network pharmacology, MAPK signaling pathway, chemokine signaling pathway

## Abstract

**Aims:** To evaluate the effectiveness and potential mechanism of calcium dobesilate (CaD) in diabetic kidney disease (DKD) patients.

**Methods:** We searched for available randomized controlled studies on DKD patients’ treatment with CaD through open databases. Continuous variables were expressed as standardized mean difference (SMD) with a 95% confidence interval (CI). The putative targets and possible pathways of CaD on DKD were analyzed by network pharmacology. Molecular docking was employed to verify the match between CaD and the target genes.

**Results:** In the meta-analysis, 42 trials were included, involving 3,671 DKD patients, of which 1,839 received CaD treatment in addition to conventional treatment, while 1,832 received conventional treatment. Compared with routine therapy, the levels of serum creatinine (Scr) and blood urea nitrogen (BUN) significantly decreased in the CaD treatment (early stage of DKD, Scr: *p* < 0.00001; BUN: *p* < 0.0001; clinical stage of DKD, Scr: *p* < 0.00001; BUN: *p* < 0.00001; kidney failure stage, Scr: *p* = 0.001; BUN: *p* = 0.004). The levels of serum cystatin C (Cys-C), urine levels of molecules reflecting kidney function (urinary albumin excretion rate (UAER) and micro glycoprotein), and inflammatory factors [hypersensitive c-reactive protein (hs-CRP)] were reduced compared with control groups, while glomerular filtration rate (GFR) was increased in patients treated with CaD for 12 weeks. CaD also showed a better effect on improving endothelial function. Network pharmacology results showed that the interaction pathway between CaD and DKD was mainly enriched in MAPK and chemokine signaling pathways. AKT1, CASP3, IGF1, MAPK8, and CCL5 might be the key targets for CaD in treating DKD.

**Conclusion:** Combination with CaD is effective and safe in patients with DKD. Inhibition of MAPK and chemokine signaling pathways might be vital in treating CaD in DKD patients.

## 1 Introduction

Diabetes mellitus (DM) has afflicted around 422 million people worldwide and has become a leading cause of morbidity and mortality (Zhang et al., 2017). Diabetic kidney disease (DKD) is one of the most severe microvascular consequences of diabetes, accounting for 30%–50% of all end-stage kidney disease patients (Qin et al., 2017). It also imposes a significant financial cost on patients, families, and society (Zhang, 2012). The severity of DKD was divided into five stages using an albuminuria-based methodology.

The interstitial space grows in the first two phases, the mesangial volume rises, and the glomerular basement membrane thickens. They are silent stages because there is no detectable microalbuminuria in clinical practice, and there are currently no effective indicators for their detection (Salem et al., 2020). The early and clinical stages are the third and fourth stages, respectively. Microalbuminuria to overt proteinuria is a sign of progressing from early stage to clinical overt diabetic kidney disease. End-stage renal failure (ESRD) is the ultimate stage (Cai et al., 2017). Because DKD is still reversible in its early phases, novel therapeutic medicines for aggressive therapy are urgently needed to avert progression to ESRD.

Calcium dobesilate (CaD) is a microcirculation-improving medication that improves hemodynamics, inhibits inflammatory responses, and suppresses interstitial fibrosis, among other things (Chen and Bai, 2017). CaD has long been utilized to treat diabetic retinopathy (DR) due to its potential to reduce oxidative stress by decreasing the activation of the p38MAPK and NF-B pathways (Liu et al., 2019; Ashraf et al., 2021). CaD’s efficacy in kidney illness (Zhang et al., 2021), chronic venous insufficiency (Ciapponi et al., 2004), thrombotic disorders (Michal and Giessinger, 1985), and various types of cardiac disease (Besirli et al., 2012) has received great attention recently. CaD considerably affects DKD in lowering the urine albumin excretion rate (Qin et al., 2017) and has been studied extensively in clinical trials (Zhou et al., 2018). There is little authoritative conclusion on the benefits and possible adverse effects due to the limited sample sizes and variable findings of currently available randomized controlled studies (RCTs) on CaD.

Some medications have a wide range of effects on humans, but they all point in the same therapeutic direction, implying that they can work on diverse targets in the same pathways. We have found that network-related approaches can be employed to emphasize our findings of drug action mechanisms in various data layers in the drug development process (Boezio et al., 2017). Based on the network pharmacology mechanism, we created a network between the putative CaD targets and the implicated gene targets of DKD. The PPI network is formed when common targets interact with each other. Furthermore, we may use GO and KEGG analyses on these proteins to identify the main pathway that plays a crucial role in CaD’s treatment of DKD.

## 2 Materials and methods

### 2.1 Meta-analysis

#### 2.1.1 Randomized controlled study preparation

The PRISMA (preferred reporting items for systematic reviews and meta-analyses) guidelines were followed in this meta-analysis (Page et al., 2021). The PubMed Database, MED-LINE, Global Health, EMBASE, EBSCOhost, Cochrane Library, China National Knowledge Internet, VIP, Wanfang, and SinoMed databases were used to find the literature. Information from well-known registries, such as Current Controlled Trials, the World Health Organization International Clinical Trials Registry Platform, the Clinicaltrials.gov trials registry, and published review papers and editorials, was considered. The search terms were “Calcium Dobesilate” and “diabetic kidney disease” or “diabetic kidney disease” or “diabetic kidney disease” or “randomized” or “double-blind trial”, with no restrictions on subheadings or language. Other likely relevant citations were found in the reference lists of all included papers, and the literature not found in the abovementioned electronic databases was manually reviewed.

#### 2.1.2 Inclusion and exclusion criteria of studies

The inclusive criteria were as follows: 1) study subjects were diagnosed as DKD according to the corresponding guidelines, 2) all patients were randomized to receive treatment with CaD and contemporary medications or contemporary medications alone, 3) sample size in each study group was ≥ 15cases, 4) follow-up in each study group was ≥ 8 weeks; and 5) the outcomes were quantitative to facilitate outcome analysis.

Exclusion criteria were as follows: 1) studies were nonrandomized or nonblinded, 2) patients enrolled had no definite diagnosis, 3) different medications were compared, 4) studies reported only symptomatic changes in patients without objective laboratory measurements; and 5) methodological quality was poor with a Jadad score <2.

#### 2.1.3 Stage of diabetic kidney disease

DKD is categorized into five stages according to the albuminuria-based classification. There was a hemodynamic alteration at the start of the first stage, with increased glomerular capillary hydrostatic pressure but no abnormalities in the ultrastructure. Hyperglycemic effects begin in the second part of the first stage, with thickening of the glomerular basement membrane, increased mesangial volume, and interstitial expansion. Because microalbuminuria cannot be measured in clinical practice and no suitable test marker has yet been discovered, the second stage is quiet (Salem et al., 2020). The early stage of diabetic kidney disease is the third stage. Previous structural changes had become severe, and microalbuminuria had been diagnosed. The fourth stage is known as clinically severe diabetic kidney disease, and it is at this stage that these changes may progress to significant proteinuria, formerly known as “macroalbuminuria.” Microalbuminuria to overt proteinuria is a marker of progression from early stage to clinical overt diabetic kidney impairment. ESRD is the final stage (Cai et al., 2017).

#### 2.1.4 Statistics analysis

RevMan version 5.3 was used to synthesize the data. Continuous variables were expressed as standardized mean difference (SMD) with a 95% confidence interval (CI). Chi-square and I2 tests were used to test heterogeneity. Nonheterogeneous results (P˃0.1, I2 < 50%) were adopted for the fixed effects model, and heterogenous results (*p* ≤ 0.1, I2 ˃ 50%) were adopted for the random effects model. Statistical significance was defined as a two-tailed *p* < 0.05. The fail-safe number was used to estimate the extent of publication bias (Cheng et al., 2016). The formula for the fail-safe number was Nfs0.05 = (ΣZ/1.64)2-S, where S is the number of the included trials.

### 2.2 Network pharmacology

#### 2.2.1 Predicting potential targets of calcium dobesilate

The bioactive component of CaD was found in the published literature using the keyword “Calcium Dobesilate” in the PubMed database. The 2D structure of CaD was then retrieved from PubChem (Kim et al., 2021) and uploaded to Pharmmapper (Wang et al., 2017) to forecast the drug’s potential targets. All target names were entered into Uniprot sites (UniProt Consortium., 2019) and selected by Homo Saipan species to standardize the gene information.

#### 2.2.2 Screening of targets for diabetic kidney disease

The target genes of DKD were found using the key phrases “diabetic kidney disease” or “diabetic kidney dis-ease” in the Online Mendelian Inheritance in Man database (OMIM) (Amberger et al., 2015), Gene Cards database (Stelzer et al., 2016), and DisGeNET database (Piñero et al., 2017).

#### 2.2.3 Construction of protein–protein interaction network

To identify the interaction targets of CaD in the treatment of DKD, we selected the online drawing tool Interactive Venn (Heberle et al., 2015) to draw a Venn diagram, whose overlapping section represented the typical targets for CaD and DKD. These common targets were uploaded to the STRING 11.0 platform (Szklarczyk et al., 2019), and the PPI network was built using the STRING database and the Network Analyzer plugin of Cytoscape (Shannon et al., 2003). The stronger the interaction in a network, the higher the degree. The more significant degree nodes, which may play a critical role in the overall network, were screened based on the network’s topological qualities.

#### 2.2.4 Enrichment analysis

Metascape^1^ (Zhou et al., 2019) was used to perform Gene Ontology (GO) functional analysis,Kyoto Encyclopedia of Genes, and Genomes (KEGG) pathway enrichment analysis, with *p* ≤ 0.01 as the cut-off threshold. Bioinformatics web tools^2^ and the EHBIO Gene Technology Platform^3^ were then used to display the top 10 GO items and 20 KEGG pathways that met the criteria.

#### 2.2.5 Construction of the component–target–pathway network

The integrated network of component–target–pathway was constructed using Cytoscape 3.7.1. The topology parameters of the network were analyzed with the built-in tool Network Analyzer in Cytoscape to identify the relationships of protein targets with components and the involved pathways.

#### 2.2.6 Molecular docking verification

**FIGURE 1:**
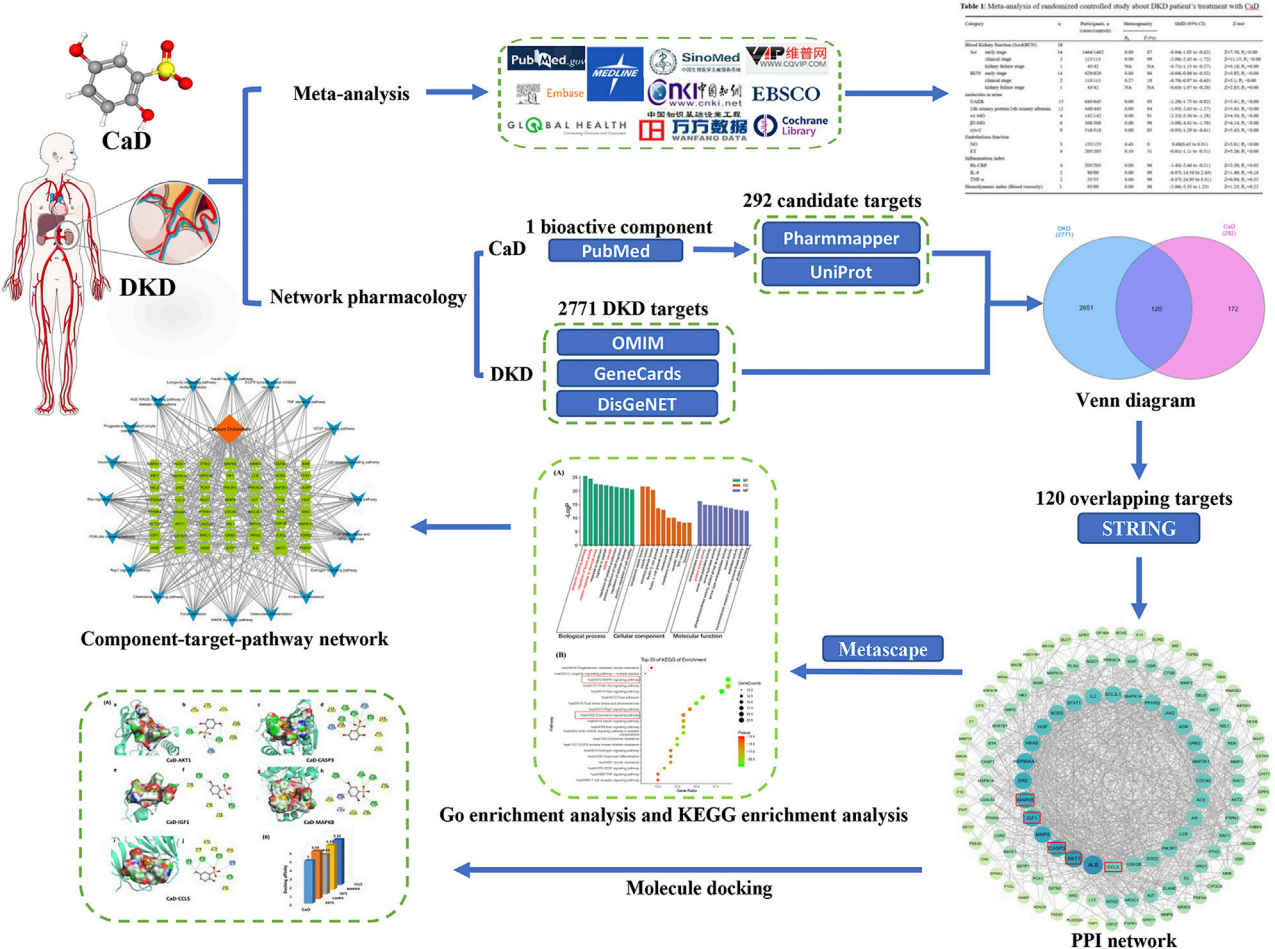
Flow chart of the investigation of calcium dobesilate in the treatment of diabetic kidney disease.

Suitable 3D structure “pdb” files of the core targets were downloaded from RCSB Protein Data Bank (Berman et al., 2000). PyMol2.6.0 embellished the downloaded complexes to remove the original ligand and water molecules. The “sdf” file of the DKD bioactive component from the PubChem database was obtained, and its format was converted to a “pdb” file by Open Babel2.4.0 (O'Boyle et al., 2011). Then, we used AutoDockTools1.5.6 (Goodsell and Olson, 1990) to evaluate and verify the binding affinity of the compound–target relationship. The critical models were visualized by PyMol2.6.0 software and Discovery Studio4.5 software. A flow chart of the meta-analysis, network pharmacology, and molecular docking is shown in Figure 1.

## 3 Result

### 3.1 Meta-analysis

**FIGURE 2:**
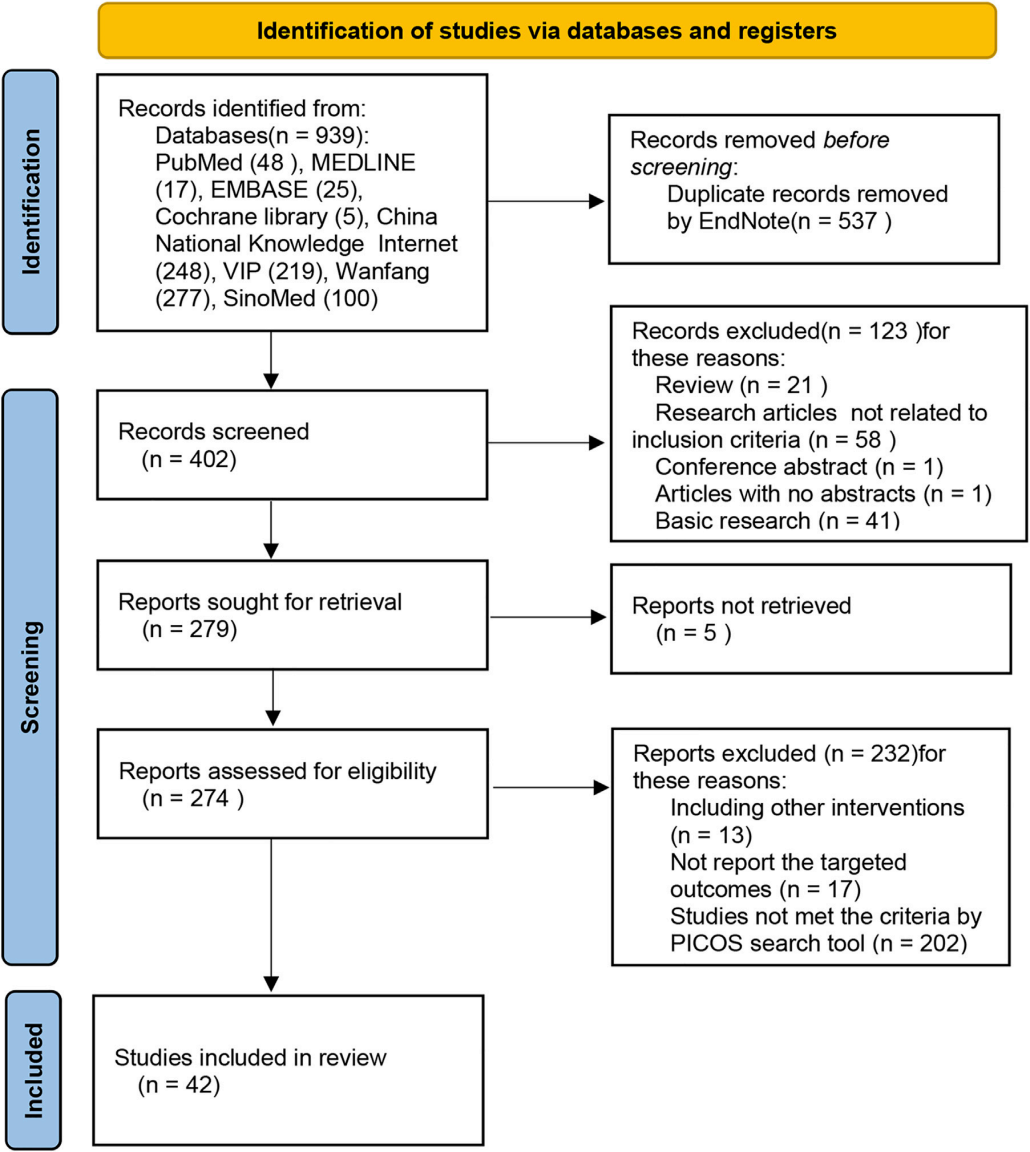
Flow chart of the systematic search process.

A total of 939 trials were retrieved through database searches—PubMed (48), MEDLINE (17), EMBASE (25), Cochrane library (5), China National Knowledge Internet (248), VIP (219), Wanfang (277), and SinoMed (100). Five hundred and thirty-seven duplicate records were eliminated using endnote software. Subsequently, among the remaining 402 pieces, 123 trials were excluded from the primary screening according to the inclusion and exclusion criteria. Of the remaining 279 trials, 273 were evaluated as eligible, and 42 shots were finally included for meta-analysis (Figure 2; Table 1).

**TABLE 1:** Meta-analysis of the randomized controlled study of DKD patients’ treatment with CaD.

Category	n	Participants, n (cases/controls)	Heterogeneity		SMD (95%CI)	Z-test
			P_h_	I^2^ (%)		
Blood kidney function (Scr&BUN)	38					
Scr early stage-all	34	1,464/1,462	0.00	87	−0.84 (−1.05 to −0.62)	*Z* = 7.58; *P*_*z*_<0.00
early stage-8W	9	500/499	0.00	78	−0.45 (−0.73 to −0.17)	*Z* = 3.16; *P*_*z*_<0.00
early stage-12W	23	898/895	0.00	89	−0.97 (−1.27 to −0.67)	Z = 6.37; *P*_*z*_<0.00
clinical stage-12W	2	113/113	0.00	99	−2.08 (−2.45 to −1.72)	*Z* = 11.15; *P*_*z*_<0.00
kidney failure stage-12W	1	43/42	NA	NA	−0.71 (−1.15 to −0.27)	*Z* = 3.18; *P*_*z*_ = 0.00
BUN early stage-all	14	629/629	0.00	86	−0.64 (−0.96 to −0.32)	*Z* = 3.92; *P*_*z*_<0.00
early stage-8W	4	281/281	0.00	0	−0.48 (−0.65 to −0.31)	*Z* = 5.60; *P*_*z*_<0.00
early stage-12W	9	297/295	0.00	87	−0.55 (−1.03 to −0.07)	*Z* = 2.24; *P*_*z*_ = 0.03
clinical stage-12W	2	113/113	0.27	19	−0.70 (−0.97 to −0.43)	*Z* = 5.1; *P*_*z*_<0.00
kidney failure stage-12W	1	43/42	NA	NA	−0.63 (−1.07 to −0.20)	*Z* = 2.85; *P*_*z*_ = 0.00
Serum Cys-C	9	518/518	0.00	85	−0.95 (−1.29 to −0.61)	*Z* = 5.43; *P*_*z*_<0.00
Serum Cys-C-8W	4	276/278	0.02	68	−0.78 (−1.10 to −0.45)	*Z* = 4.73; *P*_*z*_<0.00
Serum Cys-C-12W	5	242/240	0.00	91	−1.13 (−1.78 to −0.47)	Z = 3.37; P_z_<0.00
GFR						
GFR -8W	2	82/79	1.00	0	0.17 (−0.14–0.48)	*Z* = 1.09; *P*_*z*_ = 0.27
GFR-12W	2	82/79	0.26	22	1.66 (1.26–2.07)	*Z* = 7.99; *P*_*z*_<0.00
Molecules in urine						
UAER	15	643/645	0.00	93	−1.29 (−1.75 to −0.82)	*Z* = 5.41; *P*_*z*_<0.00
24 h urinary protein/24 h urinary albumin	12	448/443	0.00	94	−1.95 (−2.63 to −1.27)	*Z* = 5.63; *P*_*z*_<0.00
α1-MG	4	142/142	0.00	91	−2.32 (−3.36 to −1.28)	*Z* = 4.38; *P*_*z*_<0.00
β2-MG	6	368/368	0.00	98	−3.00 (−4.41 to −1.58)	*Z* = 4.14; *P*_*z*_<0.00
Endothelium function						
NO	3	155/155	0.43	0	0.68 (0.45–0.91)	*Z* = 5.81; *P*_*z*_<0.00
ET	4	205/205	0.10	51	−0.81 (−1.11 to −0.51)	*Z* = 5.26; *P*_*z*_<0.00
Inflammation index						
Hs-CRP	4	205/205	0.00	96	−1.43 (−2.66 to −0.21)	*Z* = 2.30; *P*_*z*_ = 0.02
IL-6	2	80/80	0.00	99	−6.07 (−14.56 to 2.43)	*Z* = 1.40; *P*_*z*_ = 0.16
TNF-α	2	55/55	0.00	99	−8.07 (−24.95 to 8.81)	*Z* = 0.94; *P*_*z*_ = 0.35
Hemodynamic index (Blood viscosity)	2	93/90	0.00	98	−2.06 (−5.35 to 1.23)	*Z* = 1.23; *P*_*z*_ = 0.22

*P*_*Z*_<0.05, shows a significant association. CI, confidence interval; NA, not available; SMD, standardized mean difference; Ph, *p*-values for heterogeneity of Q-test; Scr, serum creatinine; BUN, blood urea nitrogen; GFR, glomerular filtration rate; UAER, urine albumin excretion rate; a1-MG, alpha-1-microglycoprotein; β2-MG, β2-microglobulin; Cys-C, cystatin C; NO, nitric oxide; ET, endothelin; Hs-CRP, hypersensitive c-reactive protein; IL-6, interleukin-6; and TNF-α, factor-α.

#### 3.1.1 Blood kidney function (Scr&BUN)

Among all of the 42 trials, 38 trials evaluated the Scr and BUN. Thirty-five of the 38 trials were focused on DKD in its early stages. Two trials (Zhang et al., 2013; Wang et al., 2015) focused on DKD patients in the clinical phase, while one (Wu et al., 2007) concentrated on DKD patients in the kidney failure stage. As for the studies performed on the early stage of DKD patients, the results showed that the function of the kidney was markedly better in the CaD treatment group than that of the control groups (Scr: SMD = −0.84; 95% CI, −1.05 to −0.62; *p* < 0.00001; I^2^ = 87%, BUN: SMD = −0.64; 95% CI, −0.96 to −0.32; *p* < 0.0001; I^2^ = 86%). The early phase was subdivided into 8-week and 12-week treatment cycles in most of the included literature. Furthermore, the results showed that CaD treatment for 8 weeks (Scr: SMD = −0.45; 95% CI, −0.73 to −0.17; *p* = 0.002; I^2^ = 78%, BUN: SMD = −0.48; 95% CI, −0.65 to −0.31; *p* < 0.00001; I^2^ = 0%) and 12 weeks (Scr: SMD = −0.97; 95% CI, −1.27 to −0.67; *p* < 0.00001; I^2^ = 89%, BUN: SMD = −0.55; 95% CI, −1.03 to −0.07; *p* = 0.03; I^2^ = 87%) could significantly reduce Scr and BUN levels compared to the control group. In the clinical stage of DKD, patients treated with CaD for 12 weeks also exhibited a statistically significant reduction in the expression level of the Scr and BUN compared with control groups (Scr: SMD = −2.08; 95% CI, −2.45 to −1.72; *p* < 0.00001; I^2^ = 99%; BUN: SMD = −0.70; 95% CI, −0.97 to −0.43; *p* < 0.00001; I^2^ = 19%). In the kidney failure stage of DKD, the data showed the same trend, that the groups treated with CaD for 12 weeks had a better effect on reducing Scr and BUN than comparators (Scr: SMD = −0.71; 95% CI, −1.15 to −0.27; *p* = 0.001; BUN: SMD = −0.63; 95% CI, −1.07 to −0.20; *p* = 0.004).

#### 3.1.2 Serum cystatin C

Serum cystatin C (Cys-C) has been recognized as an ideal marker of kidney function (Dharnidharka et al., 2002). Nine trials (Hong, 2013; Wang et al., 2015; Cen et al., 2016; Liu et al., 2016; Chen and Bai, 2017; Fan and Ma, 2017; Qin et al., 2017; Li, 2018; and Zeng et al., 2018) reported serum Cys-C. The treatment durations were both 8 and 12 weeks. A total of 1,036 patients were included, 518 of whom were in the treatment group and 518 in the control group. The results proved that the CaD groups exhibited a statistically significant reduction in serum Cys-C compared with the control groups (SMD = −0.95; 95% CI, −1.29 to −0.61; *p* < 0.00001; I^2^ = 85%). Subgroup analysis was conducted to determine that the source of heterogeneity was from different treatment methods: one trial (Qin et al., 2017) reported the patients treated with CaD plus alprostadil and routine treatment, another (Wang et al., 2015) focused on the patients treated with CaD plus α-thioctic acid plus routine treatment, and the remaining studies (Zhang, 2012; Hong, 2013; Cen et al., 2016; Liu et al., 2016; Chen and Bai, 2017; Fan and Ma, 2017; Li, 2018) compared two treatment methods, including CaD, angiotensin-converting enzyme inhibitor (ACEI)/angiotensin II receptor blockers (ARBs) and routine treatment (CaD group) or ACEI/ARB and routine treatment (control group) (MD = −0.37; 95% CI, −0.43 to −0.30; *p* < 0.00001; I^2^ = 18%).

#### 3.1.3 Glomerular filtration rate

Glomerular filtration rate (GFR) is widely recognized as a comprehensive measure of renal function, and its assessment is essential for clinical practice, research, and public health (Levey et al., 2020). GFR was reported in two trials (Wu et al., 2007; Zhang, 2018), and both treatment cycles were divided into 8 and 12 weeks. The results revealed that after 12 weeks of treatment, patients in the CaD group had considerably higher GFR levels than the control group (SMD = 1.66; 95% CI, 1.26 to 2.07; *p* < 0.00001; I^2^ = 22%), while patients who received CaD for 8 weeks had increased GFR levels, but there was no statistical difference (SMD = 0.17; 95% CI, −0.14 to 0.48; *p* = 0.27; I^2^ = 0%).

#### 3.1.4 Urinary albumin excretion rate, 24 h urinary protein/24 h urinary albumin, α1-MG, and β2-MG

The kidney function can be quantified by detecting the level of some molecules in urine. These molecules are divided into primary outcomes and secondary outcomes. The primary outcomes include urine albumin excretion rate (UAER) and 24 h urine protein/24 h urine albumin, and the secondary outcomes consist of alpha-1-microglycoprotein (α1-MG) and β2-microglobulin (β2-MG).

##### 3.1.4.1 Urinary albumin excretion rate

Evaluated UAER, as the primary symptom of DKD, leading to a decrease of GFR and proteinuria, is regarded as the essential criterion for diagnosing DKD in the early stage (Chen et al., 2017). UAER was reported in 15 trials (Hong, 2013; Li et al., 2013; Luo and Tao, 2014; Kang et al., 2014; Huang, 2015; Wang, 2015; Gao and Zhang, 2015; Liu et al., 2016; Yu et al., 2016; Deng and Yuan, 2016; Wang, 2016; Jiang, 2017; Yang, 2018; Zeng et al., 2018; Ma and Pang, 2018). A total of 1,288 patients were tested, 643 included in the treatment group and 645 in the control group. The results proved that UAER was reduced after CaD treatment compared with control groups (SMD = −1.29; 95% CI, −1.75 to −0.82; *p* < 0.00001; I^2^ = 93%).

##### 3.1.4.2 24 h urinary protein/24 h urinary albumin

The data was reported in 12 trials (Wu et al., 2007; Sun, 2012; Zhang, 2012; Luo and Tao, 2014; Huang, 2015; Deng and Yuan, 2016; Liu et al., 2016; Zhu, 2016; Kuang, 2017; Deng, 2018; Yang, 2018; Zhang, 2008), which included a total of 891 patients, of which 448 were in the treatment group and 443 were in the control group. According to the results, we can see that the 24 h urinary protein/24 h urinary albumin of the CaD group is reduced compared with the control group (SMD = −1.95; 95% CI, −2.63 to −1.27; *p* < 0.00001; I^2^ = 94%)

##### 3.1.4.3 α1-MG and β2-MG

α1-MG and β2-MG are tubular markers and could be predictive in diagnosing DKD patients due to their accuracy (Ferguson et al., 2015). Four trials (Hong, 2013; Zheng et al., 2013; Zhao et al., 2014; Liu et al., 2015) reported α1-MG. In trials targeting α1-MG, 284 patients were included, 142 in the treatment group and 142 in the control group. Control group patients were treated with ACEI, ARB, and routine treatment. The treatment duration was all less than or equal to 12 weeks. The result showed that the CaD group had a better curative effect (SMD = −2.32; 95% CI, −3.36 to −1.28; *p* < 0.0001; I^2^ = 91%). On the other hand, six trials (Wang et al., 2015; Jiang, 2016; Chen and Bai, 2017; Fan and Ma, 2017; Qin et al., 2017; Zhou and Chen, 2017) reported β2-MG. Seven hundred and thirty-six patients were included, half in the control and half in the CaD group. We conclude by analyzing the results: CaD can effectively improve the pathological changes of the kidney caused by DKD, making the MG in urine decrease significantly (SMD = −3.00; 95% CI, −4.41 to −1.58; *p* < 0.0001; I^2^ = 98%).

#### 3.1.5 Endothelium function

Endothelin (ET) and nitric oxide (NO) were used to show endothelial function. Three trials (Wang et al., 2015; Wang, 2016; Kuang, 2017) reported NO, and four trials (Wang et al., 2015; Wang, 2016; Kuang, 2017; Zeng et al., 2018) reported ET. The treatment duration was all equal to or less than 12 weeks. The result showed that the CaD groups had a better effect on increasing NO (SMD = 0.68; 95% CI, 0.45 to 0.91; *p* < 0.00001; I^2^ = 0%) and decreasing ET (SMD = −0.81; 95% CI, −1.11 to −0.51; *p* < 0.00001; I^2^ = 51%) than the control groups.

#### 3.1.6 Inflammation index

Inflammation response was one of the mechanisms related to DKD development. By modifying vascular permeability; releasing vasodilator and vasoconstrictor mediators; causing kidney fibrosis; and inducing cytotoxicity, apoptosis, and necrosis in the pathogenesis and progression of DKD, it may have an impact on the glomerular filtration function (Zhou et al., 2018). The enhanced inflammatory markers such as tumor necrosis factor-α (TNF-α), interleukin-6 (IL-6), and c-reactive protein (CRP) could be suggestive molecules in the development of diabetic vascular disease.

##### 3.1.6.1 Hypersensitive c-reactive protein

Four trials (Chen and Bai, 2017; Fan and Ma, 2017; Qin et al., 2017; Jia et al., 2018) were collected to evaluate the Hs-CRP. Four hundred and ten patients were included in the total, half of which were in the CaD group and half in the control group. The results showed that CaD could effectively decrease the level of CRP compared with control groups (SMD = −1.43; 95% CI, −2.66 to −0.21; *p* = 0.02; I^2^ = 96%).

##### 3.1.6.2 Interleukin-6

We collected two trials (Gao and Zhang, 2015; Qin et al., 2017) involving 160 DKD patients to assess the value of IL-6. In one trial, patients in the control group were treated with alprostadil, while in the other, the control group gave patients with telmisartan. According to this result, we can infer that no significant correlation was found between the CaD and the level of IL-6 (SMD = −6.07; 95% CI, −14.56 to 2.43; *p* = 0.16; I^2^ = 99%).

##### 3.1.6.3 Tumor necrosis factor-α

Two studies (Qin et al., 2017; Jia et al., 2018) targeted the measure of TNF-α value. One hundred and ten patients were included in the total, 55 of which were in the control group. Although one trial (Qin et al., 2017) demonstrated a remarkable decrease in TNF-α in CaD patients compared to the control group, the other (Jia et al., 2018) did not exhibit a significant difference between the CaD group and control group (SMD = −8.07; 95% CI, −24.95 to 8.81; *p* = 0.35; I^2^ = 99%).

#### 3.1.7 Hemodynamic index

Blood viscosity can reflect the state of hemodynamics, and alternation of hemodynamics is one of the critical pathways in DKD pathological development. We found two studies (Gao and Zhang, 2015; Ma and Pang, 2018) estimating the value of blood viscosity, and the result showed that CaD had no significant correlation with blood viscosity (SMD = −2.06; 95% CI, −5.35 to 1.23; *p* = 0.22; I^2^ = 98%).

### 3.2 Network pharmacology

#### 3.2.1 Candidate targets of Calcium Dobesilate and diabetic kidney disease

**FIGURE 3:**
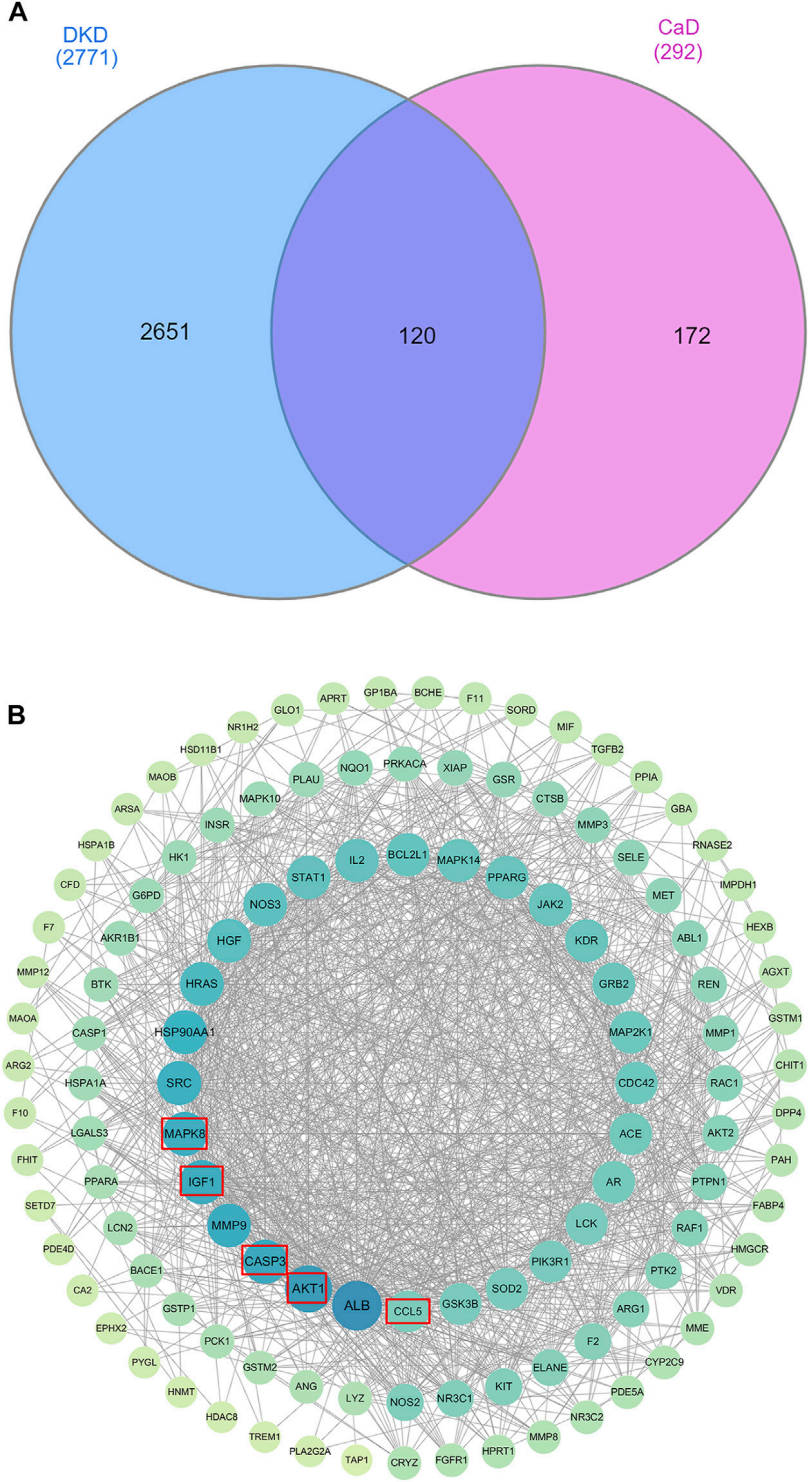
Venn diagram and PPI work. **(A)** Venn diagram. The blue section indicates DKD-related targets, and the pink section indicates CaD-related targets. Twenty-five targets in the middle overlapping section are common targets of DKD and CaD. **(B)** PPI network. A total of 119 target proteins and 1,175 interacting edges are present in the network. Sizes and colors of the nodes are illustrated from big to small and blue to green in a descending order of degree values.

For CaD, there are 293 related targets collected by searching the database. For DKD, we have retrieved 534 genes from the OMIM database, 1,678 genes from the Gene Cards database, and 1,189 genes from the DisGeNET database. After eliminating the redundancy, 2,771 known therapeutic targets were collected in this study. By drawing a Venn diagram to look for the intersection of CaD and DKD targets, 120 overlapping genes were obtained (Figure 3A).

#### 3.2.2 Protein–protein interaction network of diabetic kidney disease targets

To evaluate the role of potential targets in complicated diseases and discover connections, overlapping targets are submitted to the STRING11.0 platform to establish a PPI network. After analyzing the topology parameters of the PPI network, the 120 targets were sorted in descending order by degree and arranged in a concentric circle (Figure 3B). Degree reflected the importance of nodes by representing the number of connections between nodes and other nodes. According to the degree, the targets in the innermost circle were predicted as important targets.

#### 3.2.3 Gene ontology and Kyoto Encyclopedia of Genes and Genomes enrichment Analyses

**FIGURE 4:**
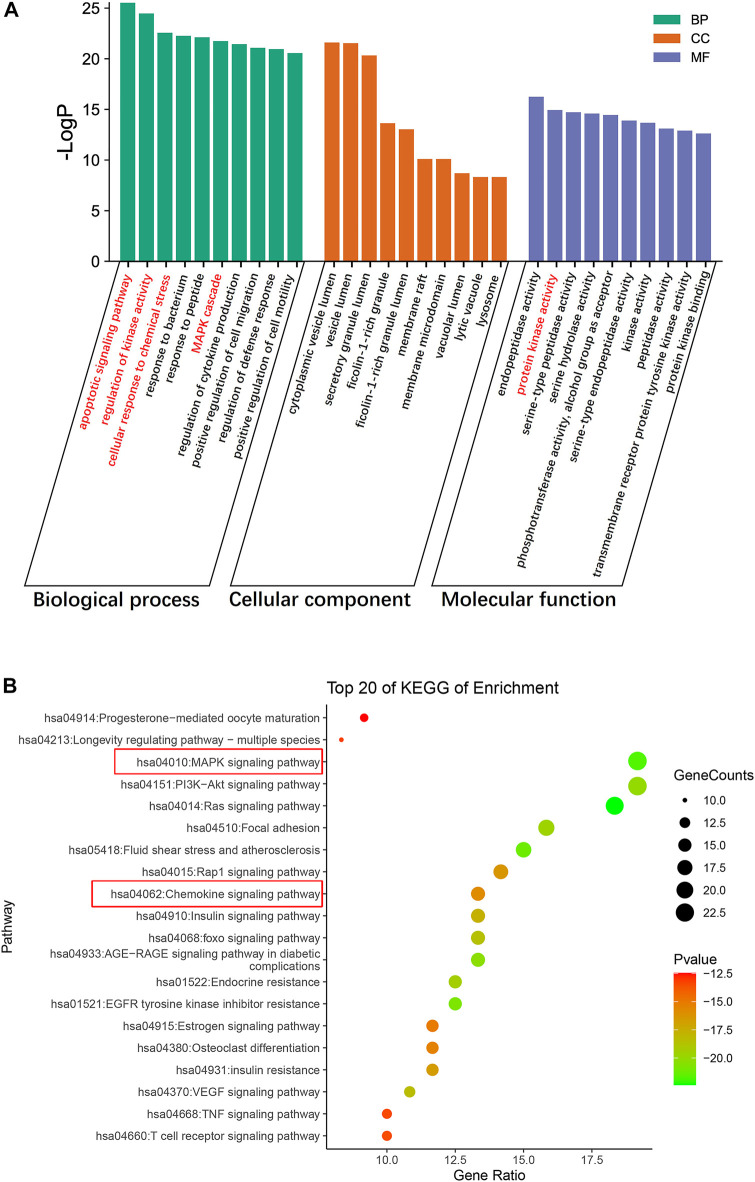
Enrichment analysis of the targets of CaD in treating DKD. **(A)** GO functional analysis. Top 10 items of each part are shown. **(B)** KEGG pathway enrichment analysis. The sizes of the bubbles are illustrated from big to small in a descending order of the number of potential targets involved in the pathways.

We conducted GO functional analysis and KEGG pathway enrichment analysis to elucidate the biological impacts of CaD on gene functions and signaling pathways of relevant targets in treating DKD. As shown in Figure 4, the top 10 GO items and top 20 KEGG pathways were selected based on the *p*-value.

For biological processes, the targets were mainly enriched in the apoptotic signaling pathway, regulation of kinase activity, MAPK cascade, cellular response to chemical stress, and regulation of cytokine production. The top 10 items were mainly related to protein kinase activity, peptidase activity, and phosphotransferase activity (alcohol group as acceptor) in molecular functions. The cellular components were primarily concentrated in cytoplasmic vesicle lumen, secretory granule lumen, ficolin-1-rich granule lumen, membrane raft, vacuolar lumen, lytic vacuole, and lysosome; according to the results of the KEGG pathway enrichment analysis, most were involved in the MAPK signaling pathway, PI3K-Akt signaling pathway, the Ras signaling pathway, focal adhesion, fluid shear stress, atherosclerosis, Rap1 signaling pathway, and the chemokine signaling pathway. Interestingly, the typical targets of CaD and DKD are mainly enriched in the MAPK signaling pathway, and protein kinase activity was mentioned in the top 10 molecular function items. Additionally, biological processes involved apoptosis signaling pathways, regulation of kinase activity, and MAPK cascade. Therefore, we surmised that the MAPK signaling pathway might be critical. The number of targets concentrated in the chemokine pathway was relatively large in the figure, and the biological process highlighted cellular response to chemical stress and regulation of cytokine production. Meanwhile, the meta-analysis results showed that CaD significantly reduced the level of inflammatory factors. These imply that the chemokine signaling pathway cannot be overlooked.

#### 3.2.4 Component–target–pathway network construction

**FIGURE 5:**
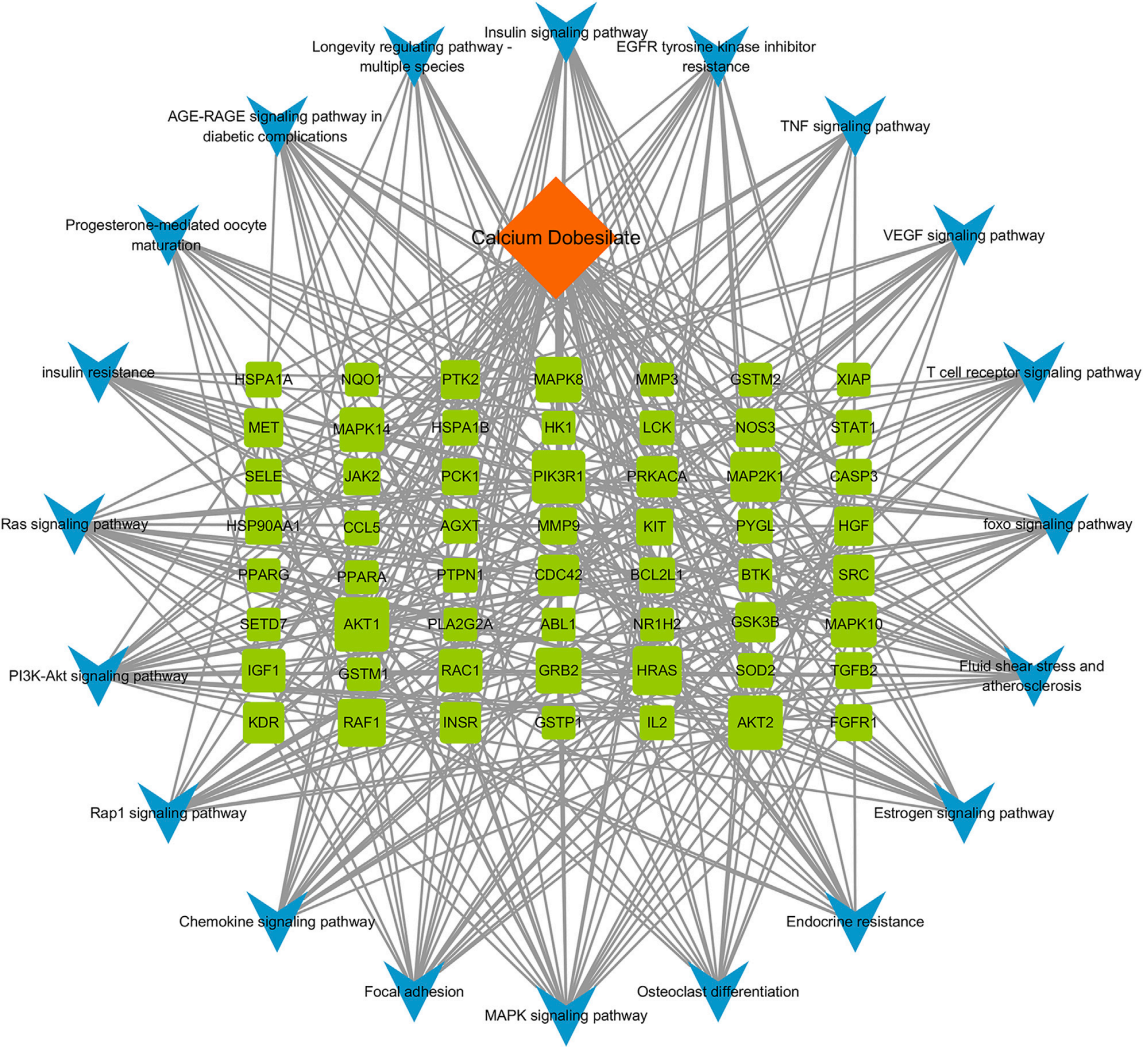
Component–target–pathway network. A total of 77 nodes and 372 edges are present in the network. Orange diamond represents the bioactive component of CaD, 57 green squares represent targets, and 20 blue V-shapes represent pathways. Sizes of the green square node are illustrated from big to small in a descending order of degree values. A total of 372 edges represent the interaction relationship between components, targets, and pathways.

A component–target–pathway network was constructed with Cytoscape3.7.1 based on the KEGG pathway enrichment analysis (Figure 5). Among the essential targets selected by the PPI network, AKT1, CASP3, IGF1, and MAPK8 were enriched in the MAPK signaling pathway, and CCL5, a typical inflammatory chemokine, was increased in the chemokine signaling pathway at the same time. We speculated that the five targets might be critical for CaD in treating DKD and used for further molecular docking.

#### 3.2.5 Molecular docking result analysis

**FIGURE 6:**
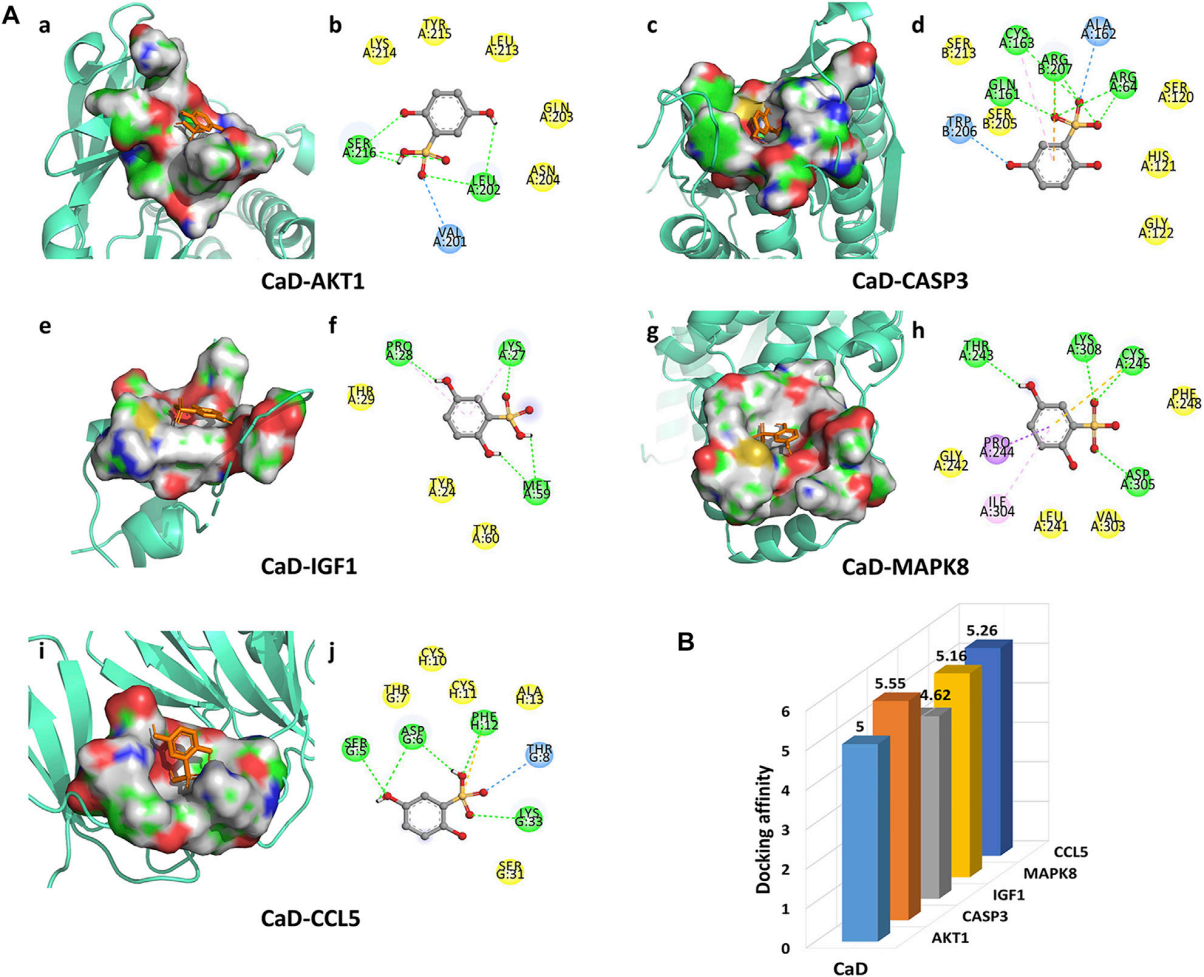
Molecular docking diagram. **(A)** Five conformations of a molecular docking simulation. Diagrams (3D) represent that the molecular model of the compound is in the binding pocket of the protein. The compound is shown as a stick model in orange. The amino acid residues in the surrounding are represented by surface style. Diagrams (2D) show the interactions between the compound and surrounding residues. **(B)** 3D column diagram shows the affinity of six conformations. *X*-axis: bioactive component, *y*-axis: target names, and *z*-axis: docking affinity (absolute value of the binding energy).

In the present studies, the possible interaction activity between five key targets and their corresponding compounds of CaD was investigated with molecular docking verification. Among the docking results, most binding complexes possessed high binding affinity, averaging −5.12 kcal/mol. The modes of five binding complexes are displayed in Figure 6, including CaD-AKT1 docking (−5 kcal/mol), CaD-CASP3 docking (−5.55 kcal/mol), CaD-IGF1 docking (−4.62 kcal/mol), CaD-MAPK8 docking (−5.16 kcal/mol), and CaD-CCL5 docking (−5.26 kcal/mol). For concreteness, taking the CaD-AKT1 docking as an example, small-molecule ligand CaD may be embedded in the interfaced pocket formed by the interaction of amino acid residues in the protein (Figure 6Aa). Figure 6Ab shows three hydrogen bond formations between ligand and residues in SER 216, LEU202, and VAL201. The other essential residues (LYS214, TYR215, LEU213, GLN203, and ASN204) interacted with CaD through van der Waals forces. These forms of hydrogen bonds and interactions contribute to the stability of the binding of small molecules to the active sites of proteins.

## 4 Discussion

We gathered 42 trials of CaD intervention for DKD clinical treatment, including 3671 DKD patients in various phases of the disease, and divided them into the CaD experimental and control groups. The results showed that Scr, BUN, and Cys-C levels in the blood, as well as molecules including UAER, 24 h urine protein/24 h urine albumin, 1-MG, and 2-MG in the urine, were significantly lower in the CaD group compared to the control group, while GFR levels were substantially higher. These findings suggested that CaD could help DKD patients improve their glomerular filtration performance and kidney function. In addition, CaD could modulate endothelium contraction and relaxation by boosting NO and lowering ET, regulating microvasculature function. Inflammatory variables such as CRP levels also dropped in the CaD group, implying that CaD can help DKD patients lessen their inflammatory response. Compared to the control group, patients in the CaD group showed a trend of improvement in numerous indices, including glomerular filtering performance, endothelium function, and inflammatory function. As a result, we hypothesize that CaD may bind to multiple targets *in vivo*, activating or inhibiting multiple metabolic and disease-related pathways, reminding us that a network pharmacological approach can be used to investigate the mechanisms by which drugs improve multiple metrics in patients.

**FIGURE 7:**
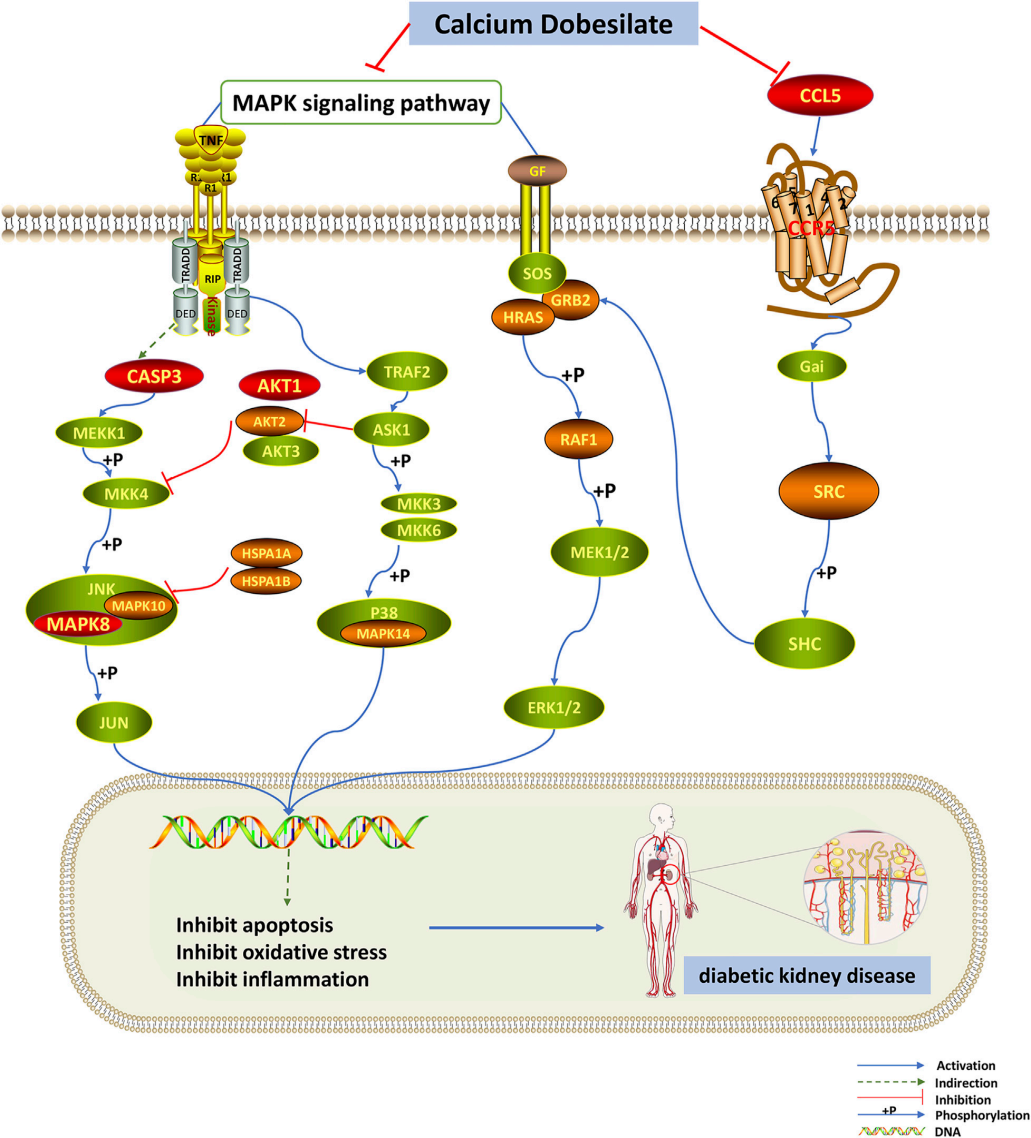
MAPK and chemokine signaling pathways are influenced by dapagliflozin. The red nodes represent the key targets, the orange nodes represent common targets of CaD and DKD targets, and the green nodes represent the other targets of these two pathways. CaD affects the phosphorylation of MAPK14 in the p38/MAPK pathway. In the JNK pathway, CaD affects the phosphorylation of MAPK8 and MAPK10 and it also indirectly affects the activation of CASP3. As for the classical MAPK signaling pathway, CaD affects the activation of GRB2, HRAS, RAF1, and the phosphorylation of MEK and ERK. The chemokine signaling pathway is closely related to the classical MAPK signaling pathway. CaD inhibits the expression of CCL5. CaD inhibits apoptosis, oxidative stress, and inflammation by inhibiting the MAPK signaling pathway and the chemokine signaling pathway, thereby exerting a therapeutic effect on DKD.

Then, using network pharmacology, we built a PPI network for the drug’s and disease’s shared targets and ran GO and KEGG analyses on these genes. According to BP enrichment, multiple genes are abundant in apoptosis, regulation of kinase activity, and cellular response to chemical stress, which could be CaD’s primary approach to suppressing the development of DKD. Based on the KEGG pathway enrichment analysis and literature research, we anticipated that CaD would play a therapeutic function in DKD primarily by modulating the MAPK signaling pathway and the chemokine signaling pathway (Figure 7). We identified AKT1, CASP3, IGF1, MAPK8, and CCL5 as primary targets based on the component–target–pathway network analysis, implying that the CaD-mediated DKD treatment is mainly related to the abovementioned targets.

Reactive oxygen species (ROS), the products of normal metabolism and xenobiotic exposure, are significant factors related to the pathogenesis of DKD (Bahmani et al., 2016; Miranda-Díaz et al., 2016), and their production can induce apoptosis (Xu et al., 2017). CaD has been shown to prevent cell apoptosis by lowering the number of ROS (Iriz et al., 2008). CASP3 is an essential component of apoptosis and is closely associated with the progression of DKD (Zhou et al., 2021). Hyperglycemia stimulates CASP3 cleavage and DNA breakage, and the resulting apoptosis leads to mesenchymal cell loss in DKD. (Mishra et al., 2005). One of the earliest events in DKD is glomerular thickening resulting from mesangial cell hypertrophy, and it has been determined that Akt signaling contributes to thylakoid hypertrophy in DKD. (Mahimainathan et al., 2006). DKD is associated with the loss of renal cells, particularly glomerular podocytes, which form the glomerular filtration barrier, and changes in Akt signaling as a critical event in podocyte loss during early DKD. In addition, inhibition of Akt and its downstream targets such as mTOR may provide future therapeutic benefits for treating DKD (Heljić and Brazil, 2011). IGF1 is an important growth factor that maintains the structure and function of nephritis and plays a vital role in the pathology of DKD (Bach and Hale, 2015). Previous studies have shown that IGF1 overexpression causes many histopathology changes such as kidney tissue hyperplasia, renal cell proliferation, nephromegaly, mesangial expansion, and increased inflammatory cytokines (Li et al., 2018). Donath also found that inhibition of IGF1R could reduce inflammation in DKD more effectively (Donath, 2013). Animal and human kidney biopsy studies have shown that activation of stress-activated protein kinases (p38 MAPK and JNK) is associated with the progression of inflammation and injury in multiple forms of kidney disease (Adhikary et al., 2004). In addition, pharmacological inhibitors of p38 MAPK or c-Jun N-terminal kinase (JNK) are effective in animal models of renal disease when used as prophylactic agents to prevent injury development or as interventional therapies to inhibit the progression of established injury (Tesch et al., 2016). These findings support the notion that p38 MAPK and JNK signaling are important therapeutic targets for preventing kidney damage. Besides, CCL5 is involved in the pathogenesis of diabetic kidney injury as an inflammatory chemokine upregulated in response to the metabolic and hemodynamic characteristics of the diabetic environment (Pérez-Morales et al., 2019). Subsequently, the desired molecular docking results strongly prove the criticality of the above five targets.

Podocytes are terminally differentiated cells, and podocyte damage is the critical event leading to proteinuria in DKD (Miranda-Díaz et al., 2016). ROS is essential for initiating podocyte apoptosis (Zhang et al., 2015). Activating p38 MAPK, a pro-apoptotic signaling factor downstream of ROS leads to cell apoptosis (Finkel and Holbrook, 2000). Evidence has shown that the increase of ROS could activate profibrotic factors, including TGF-β through P38-MAPK, which could promote the synthesis of type IV collagen (the main component of extracellular matrix (ECM)) and fibrin connection protein, thus causing an increase in ECM, and forming early DKD (Sakai et al., 2005). Previous studies have shown that leptin can signal through the leptin receptor isoform to stimulate glomerular endothelial cell proliferation, increase TGF-β1 synthesis, and type IV collagen production (Wolf and Ziyadeh, 2006). Leptin promotes fibrosis primarily on the glomerulus but can potentially prevent/reverse renal injury by normalizing metabolic disturbances, including hyperglycemia and hyperlipidemia (Suganami et al., 2005). Bulent O et al. suggested the potential importance of leptin in regulating glucose homeostasis and its possible direct application in treating disorders of glucose homeostasis (Yildiz and Haznedaroglu, 2006). These also provide help to broaden our treatment ideas. Furthermore, the MAPK pathway can be activated by chronic hyperglycemia, resulting in a local inflammatory response (Fang et al., 2012). Targeted therapy for inhibiting the p38 MAPK signaling pathway has shown preventive effects on streptozotocin-induced DKD (Lin et al., 2018). These findings indicate that CaD may significantly influence DKD by inhibiting the MAPK signaling pathway.

In addition tocell apoptosis, chemokine production has also been believed to play an essential role in the process of DKD (Dieter et al., 2019). The proinflammatory chemokine (C–C motif) ligand 12 caused glomerular sclerosis in T2D mice, and blocking its expression had a protective effect on DKD (Darisipudi et al., 2011). Inflammatory CCL5 is expressed in various cell types, including fibroblasts and renal tubular epithelial cells. Previously, upregulated CCL5 was found in the kidney, and its expression is directly related to the proteinuria concentration in kidney tubular cells (Navarro-González et al., 2011; Zhang et al., 2016). Consequently, it can be concluded from the existing research that the chemokine signaling pathway plays a vital role in CaD in treating DKD.

Although our results revealed the advantages of CaD for DKD, limitations did remain in the analysis. In the present study, we chose serum creatinine as a marker to assess renal function. At the same time, the literature has reported that CaD interferes to some extent with serum creatinine measured by sarcosine oxidase, both *in vivo* and *in vitro* (Zhou et al., 2018). However, we also evaluated serum Cys-C, which may be more sensitive for assessing renal function. Its levels are not interfered with by CaD, and many studies suggest using serum Cys-C to evaluate renal function in patients receiving CaD therapy (Guo et al., 2015). Other studies point out that microalbuminuria is the current gold standard for predicting and detecting diabetic kidney disease (Salem et al., 2020). The evaluation of urine albumin excretion rate (UAER) and 24 h urine protein/24 h urine albumin are covered in our article. Therefore, this issue is not sufficient to affect our findings.

## 5 Conclusion

In conclusion, our research systematically elucidated the underlying molecular mechanisms by which CaD interfered with DKD based on the meta-analysis, network pharmacology, and molecular docking. We predicted five key targets from complex networks and concluded that CaD exerts a therapeutic effect on DKD by inhibiting MAPK and the chemokine signaling pathway. We expect this research to provide additional reference directions for CaD as a medicine in treating diabetic microangiopathy. Further exploration can be done in the future based on this study.

## Data Availability

The original contributions presented in the study are included in the article/Supplementary Material; further inquiries can be directed to the corresponding authors.

## Footnotes

1
https://metascape.org/.

2
http://www.bioinformatics.com.cn/.

3
http://www.ehbio.com/ImageGP/.
